# Asparaginase-Induced Hypertriglyceridemia Presenting as Pseudohyponatremia during Leukemia Treatment

**DOI:** 10.1155/2014/635740

**Published:** 2014-10-27

**Authors:** Ashley Hinson, Dorothee Newbern, Corinne M. Linardic

**Affiliations:** ^1^Department of Pediatrics, Duke University Medical Center, Box 102382, Durham, NC 27710, USA; ^2^Department of Pediatrics, Phoenix Children's Hospital, 1919 E. Thomas Road, Phoenix, AZ 85016, USA; ^3^Department of Pharmacology and Cancer Biology, Duke University Medical Center, Box 102382, Durham, NC 27710, USA

## Abstract

Asparaginase is a chemotherapeutic agent used to induce disease remission in children with acute lymphoblastic leukemia (ALL). We describe the cases of two females with ALL who developed pseudohyponatremia as a presentation of hypertriglyceridemia following asparaginase treatment. Nine similar published cases of asparaginase-induced hypertriglyceridemia and its complications are also discussed. Possible mechanisms of action include inhibition of lipoprotein lipase, decreased hepatic synthesis of lipoprotein, and increased synthesis of VLDL. Effects of asparaginase-induced hypertriglyceridemia range from asymptomatic to transaminasemia, pancreatitis, and life-threatening thrombosis or hyperviscosity syndrome. All cases of hypertriglyceridemia described resolved following cessation of asparaginase treatment ± further treatments.

## 1. Introduction

Asparaginase is a chemotherapeutic agent used in most remission induction protocols for children with acute lymphoblastic leukemia (ALL). It inhibits protein synthesis by depleting cellular pools of the nonessential amino acid asparagine. Normal cells can synthesize asparagine* de novo* via the enzyme asparagine synthase, which malignant lymphoid cells lack [1]. Despite its benefits in the treatment of ALL, asparaginase can have many adverse effects, including hypertriglyceridemia and hypercholesterolemia [2]. Here we present the cases of two children whose hyperlipidemia manifested as pseudohyponatremia. We then review the mechanisms by which asparaginase may cause hyperlipidemia and discuss implications for evaluation and treatment.

## 2. Case Reports

### 2.1. Patient 1

An 11-year-old female presented with leg pain, fever, and streptococcal sepsis. A complete blood count (CBC) and peripheral blood smear revealed pancytopenia and lymphoblasts. Bone marrow evaluation showed pre-B-cell ALL. Liver function tests were slightly elevated and triglycerides (TGs) were within normal limits. She began induction chemotherapy per protocol CCG-1961 and completed treatment without complication. At a 22-month off-therapy visit, she was found to have relapsed ALL, and she began reinduction chemotherapy per protocol COG-AALL0433, which included prednisone 13.3 mg/m^2^/dose TID × 28 days, as well as intermittent vincristine, doxorubicin, PEG-asparaginase 2500 units/m^2^, and intrathecal cytarabine and methotrexate. During Induction 2, she developed hyponatremia (Na 129 mmol/L, normal values, 135–145). She was day three of five of scheduled cyclophosphamide and etoposide; it was presumed that her hyponatremia was due to cyclophosphamide-associated SIADH and that she might benefit from diuresis. However, that morning a vial of her blood appeared milky. A fasting lipid panel revealed marked increases in total cholesterol (659 mg/dL, normal <180) and TG (3636 mg/dL, normal <110) and reductions in serum HDL (<5 mg/dL, normal >40). LDL (86 mg/dL) was normal. Serum ALT and bilirubin levels were elevated but amylase and lipase were within normal limits, and she had no abdominal pain or vomiting. Correcting for the hypertriglyceridemia (corrected sodium = measured sodium + 0.2x triglycerides in g/L), her serum sodium concentration was actually normal (135 mmol/L), with no medical intervention necessary. The patient had no family history of early onset hyperlipidemia or hypertriglyceridemia, nor physical exam findings of hyperlipidemia such as xanthomas. She did have a history of insulin-dependent diabetes, but her blood glucose during this period ranged from 60 to 158 mg/dL (normal 70–140 mg/dL). She had received PEG-asparaginase one month prior, but lipid values were not checked at that time. Her TG levels decreased to <500 mg/dL at 30 days and 200 mg/dL at 90 days without medical intervention (Table 1). Serum ALT and bilirubin also declined rapidly to near-normal levels.

### 2.2. Patient 2

A 3-year-old female presented with a one-month history of fatigue and pallor. CBC revealed anemia and neutropenia. Bone marrow evaluation revealed pre-B-cell ALL, and she began induction chemotherapy per protocol COG-AALL0331, including dexamethasone 3 mg/m2/dose BID × 28 days, intermittent vincristine, PEG-asparaginase (2500 units/m^2^ on day 8), and intrathecal cytarabine and methotrexate. On day 29 of induction, she was found to have hyponatremia (121 mmol/L), with severe hypertriglyceridemia (3237 mg/dL) and marked increases in total (1209 mg/dL) and LDL (1108 mg/dL) cholesterol, while HDL levels were normal (40–89 mg/dL). AST, ALT, and bilirubin were elevated, while amylase and lipase were normal. Given her mixed hyperlipidemia, the following formula was used to calculate a corrected sodium value: corrected serum sodium = measured sodium + total lipids in mmol/10. (Total lipids = mmol cholesterol (measured chol/39) + mmol TG (measured TG/89)) [3]. The resulting value was 127 mmol/L, indicating some component of true hyponatremia in addition to pseudohyponatremia. Her cortisol and thyroid levels were normal. TG levels decreased to normal (124 mg/dL) within a week after completing induction. She did not develop complications related to her hypertriglyceridemia and as it resolved, her measured sodium, AST, ALT, and bilirubin levels also normalized (Table 2).

## 3. Discussion

### 3.1. Triglyceride-Induced Pseudohyponatremia

True hyponatremia is defined as a low serum sodium value associated with hypotonic extracellular fluid, which under extreme cases can cause cellular edema. Pseudohyponatremia, on the other hand, reflects a falsely low serum sodium value. Normally, serum consists of aqueous (93%) and nonaqueous (7%) phases, with sodium residing in the aqueous phase and glucose and lipids and proteins residing in the nonaqueous phase [3]. When serum proteins or lipids are elevated, as with patients 1 and 2, the relative fraction of nonaqueous serum becomes increased. Since serum sodium is usually measured via an indirect ion-selective electrode method, which requires dilution assuming a 93% aqueous phase, falsely low sodium values can result. Alternative methods include direct potentiometry or ultracentrifugation; in addition, formulas exist to correct for the reduced aqueous phase [4]. In Patient 1, the falsely low serum sodium reflected an increased nonaqueous phase of serum due to elevated TG-VLDL. In Patient 2, the elevated LDL (most likely secondary to her concurrent steroid therapy) and TG-VLDL both contributed to pseudohyponatremia. Pseudohyponatremia must be recognized promptly, because if treated as true hyponatremia (with either sodium repletion or water restriction), it can lead to serious clinical complications. Instead, resolution rests in finding the source of elevated proteins, glucose, or lipids in the nonaqueous phase of the patient's serum and treating it appropriately.

### 3.2. Role of Corticosteroids in Lipid Elevation

Glucocorticoids are a key component of leukemia treatment and can alter lipid profiles by increasing hepatic cholesterol synthesis. When monitoring for toxicity and making treatment decisions, it is important to determine whether hypertriglyceridemia in children undergoing ALL therapy is due to steroids, asparaginase, or both. Cremer et al. [5] examined lipid profiles in two groups of ALL patients. Group 1 received prednisone and asparaginase concurrently and showed elevations in total cholesterol, TG, chylomicron TG, *α*-cholesterol, and A1 apoprotein. Group 2 received prednisone first, followed by asparaginase alone, and showed elevations in *α*-cholesterol and A1 apoprotein during the steroid phase, but elevations of total and chylomicron TG during asparaginase only [5]. In Parsons et al. [6], steroids were given during both induction and intensification phases, while asparaginase was given during the intensification phase only. TG elevations were only observed during the intensification phase. So, hypercholesterolemia and hypertriglyceridemia seen in ALL patients are attributed to steroids and asparaginase, respectively.

### 3.3. Mechanism of Asparaginase-Associated Hypertriglyceridemia

TGs derive from two sources: exogenously from the diet as chylomicrons and endogenously from hepatic VLDL synthesis. Increased TG levels result from decreased clearance or increased synthesis of TG-rich particles [6, 7].

#### 3.3.1. Inhibition of Lipoprotein Lipase

TGs are cleared from the circulation by endothelial cell lipoprotein lipase (LPL), which catabolizes TG-rich particles (chylomicrons and VLDL) into fatty acids, and then taken up by adipose tissue. Conversely, decreased LPL activity causes elevated serum TGs [7]. Hoogerbrugge et al. [8] describe a 13-year-old girl with ALL who developed dramatic hypertriglyceridemia (9115 mg/dL) following asparaginase treatment. In measuring her LPL activity, they noted that while it increased during corticosteroid treatment, it decreased following asparaginase treatment, simultaneously with an increase in TG levels from 4.5 mmol/L (398 mg/dL) to 31.7 mmol/L (2805 mg/dL). However, when steroids and asparaginase were given concomitantly, the corticosteroid-induced rise in LPL activity was blunted [8]. Decreased asparaginase-induced LPL activity may be due to a global decrease in liver protein synthesis, as shown by decreased albumin and fibrinogen concentrations following asparaginase treatments [5, 9].

#### 3.3.2. Increased VLDL Synthesis

Increased synthesis of TG-rich particles may be another explanation for increased TG levels. Parsons et al. observed a large increase in VLDL during asparaginase treatment in patients with TG values >400 mg/dL (70.2 to 222; *P* = 0.005) as compared with pretreatment values. They also measured an increase in Apo-B100 (contained within VLDL particles) during asparaginase therapy, further suggesting an increase in the production of VLDL as a mechanism of hypertriglyceridemia [6].

### 3.4. Effects of Transient Hypertriglyceridemia following Asparaginase

Asparaginase-induced hypertriglyceridemia occurs in 10–50% of children being treated for ALL [5, 6, 10]. No predisposing risk factors, dose effect, leukemia risk group, blood glucose level, gender, age, or preparation of asparaginase correlate with magnitude of TG increase. The hypertriglyceridemia is transient, with TG levels returning to normal following treatment [8, 10–13]. Our literature search identified nine case reports of patients with elevated TG values following asparaginase therapy. Of these patients, complications included sagittal sinus thrombosis, acute pancreatitis, transaminasemia, and hyperviscosity syndrome (Table 3) [11–18]. Larger studies have revealed that complications, however, are rare. Steinherz examined lipid profiles of ALL patients treated with asparaginase and identified five patients with marked hyperlipidemia (TG level >1000 mg/dL); none of these patients experienced complications [11]. Cohen et al. evaluated cholesterol in 65 ALL patients: 18 (43%) with TG 200–400 mg/dL, three (7%) had levels 400–600, four (10%) had levels 600–1000, and five (12%) had levels >1000 mg/dL. One patient with TG >400 mg/dL experienced complications (a left sagittal sinus thrombosis and left frontal lobe infarct) [10]. In Parsons et al., none of the patients with TG levels >400 mg/dL or >1000 mg/dL developed complications [6].

### 3.5. Treatment Options

There is no consensus on the optimal treatment for asparaginase-induced hypertriglyceridemia, besides cessation of asparaginase. Current therapies target the complications (Table 3) and include low-fat diets, fibrate therapy, heparinization, and/or plasmapheresis. Cohen et al proposed a treatment strategy for asymptomatic patients with elevated TG levels detected on routine monitoring: if TG levels >400 mg/dL they were started on a low fat, low carbohydrate diet; if TG levels >600 mg/dL they fasted and were hydrated intravenously; if platelets >30,000/mm^3^, heparin was started. Finally, patients with TG levels >1000 mg/dL started fibrate therapy, which was discontinued after TG decreased to <300 mg/dL (usually within weeks of therapy initiation). None of these patients developed complications of their hypertriglyceridemia [10].

### 3.6. Closing and Future Directions

Asparaginase is a critical component of ALL treatment but is associated with multiple potential toxicities, including hypertriglyceridemia. We present two cases of hypertriglyceridemia presenting as pseudohyponatremia. Awareness of this metabolic side effect is imperative and could be life-saving. If not interpreted correctly, inappropriate interventions may occur (as in our cases where hyponatremia was initially thought to be due to SIADH caused by concomitant chemotherapy agents). Further studies on patient risk factors may help plan lipid monitoring protocols or guide therapies for lipid elevations.

## Figures and Tables

**Table 1:** Patient 1 laboratory values before, during, and after asparaginase treatment.

Laboratory test	Before Asparaginase	During Asparaginase	Following Asparaginase
Sodium (mmol/L)	139	129	137
Total cholesterol (mg/dL)	185	659	159
Triglycerides (mg/dL)	159	3636	204
HDL (mg/dL)	40	<5	45
LDL (mg/dL)	96	86	86
Amylase (U/L)	36	36	38
Lipase (U/L)	58	19	34
Total bilirubin (mg/dL)	0.6	1.6	0.8
Alkaline phosphatase (U/L)	167	158	105
AST (U/L)	66	n/a	45
ALT (U/L)	56	332	54

HDL: high density lipoprotein; LDL: low density lipoprotein; AST: aspartate aminotransferase; ALT: alanine aminotransferase.

**Table 2:** Patient 2 laboratory values before, during, and after asparaginase treatment.

Laboratory test	Before Asparaginase	During Asparaginase	Following Asparaginase
Sodium (mmol/L)	136	121	140
Total cholesterol (mg/dL)	n/a	1209	179
Triglycerides (mg/dL)	n/a	3237	75
HDL (mg/dL)	n/a	89	70
LDL (mg/dL)	n/a	1108	95
Amylase (U/L)	43	104	89
Lipase (U/L)	15	47	24
Total bilirubin (mg/dL)	0.4	3	0.8
Alkaline phosphatase (U/L)	167	176	105
AST (U/L)	45	202	33
ALT (U/L)	19	188	37

HDL: high density lipoprotein; LDL: low density lipoprotein; AST: aspartate aminotransferase; ALT: alanine aminotransferase; n/a: not available.

**Table 3:** Case reports in the English literature of asparaginase-induced hypertriglyceridemia.

Age	Sex	Disease	Phase of therapy	Lipid level	Complications	Treatment	Resolution	Ref
9	F	T cell ALL	N/a	N/a	Sagittal sinus thrombosis, transient diabetes mellitus	N/a	N/a	[12]

10	F	Pre-B-cell ALL	Consolidation II	Trig: 20,600 mg/dL Chol: 1640 mg/dL	Lipemia retinalis, moderate transaminasemia	Heparinized	Returned to nml 2 weeks after completion of asp treatment	[11]

10	F	Pre-B-cell ALL	Induction	Trig: 1817 mg/dL Chol: 1116 mg/dL	Weakness in lower extremities, lethargy c/w hyperlipidemia-associated hyperviscosity syndrome, moderate transaminasemia	Initiation of gemfibrozil and omega-3 marine oil	Improvement in lipid levels and liver function tests with lipid-lowering drugs	[18]

10	M	T cell ALL	Induction	Trig: 4040 mg/dL Chol: 540 mg/dL	Acute pancreatitis	Plasmapheresis, removal of asp from treatment plan, low fat diet	Nml within 15 days	[17]

10	F	Pre-B-cell ALL	Maintenance	Trig: 2700 mg/dL Chol: 1135 mg/dL	Lipemia retinalis	Heparinized	Nml	[13]

13	F	Pre-B-cell ALL	Induction	Trig: 103 mmol/L Chol: 7.6 mmol/L	N/a	N/a	N/a	[8]

16	F	Pre-B-cell ALL	Induction	Trig: 8510 mg/dL Chol: 660 mg/dL	Lipemia retinalis, moderate transaminasemia, lethargy, anginal pain, dyspnea	Plasmapheresis, heparinized, acipimox/olbetam antilipidemic	Rapid nml	[15]

17	F	Pre-B-cell ALL	Reinduction	Trig: 5250 mg/dL Chol: 672 mg/dL	Mild abdominal pain	Conservative management	Nml within 3 weeks	[16]

18	M	ALL	End of Consolidation I	Trig: 1742 mg/dL	Acute pancreatitis	Conservative management	Nml within 2 weeks	[14]

F: female; M: male; Trig: triglycerides; Chol: cholesterol; C/w: consistent with; Nml: normal; Asp: asparaginase; N/a: not available.

## Abbreviations

ALL: Acute lymphoblastic leukemia
HDL: High density lipoprotein
LDL: Low density lipoprotein
LPL: Lipoprotein lipase
TGs: Triglycerides
VLDL: Very low density lipoprotein.
